# UV‐induced DNA Damage in Skin is Reduced by CaSR Inhibition

**DOI:** 10.1111/php.13615

**Published:** 2022-03-29

**Authors:** Chen Yang, Mark Stephen Rybchyn, Warusavithana Gunawardena Manori De Silva, Jim Matthews, Andrew J. A. Holland, Arthur David Conigrave, Rebecca Sara Mason

**Affiliations:** ^1^ Department of Physiology and Bosch Institute School of Medical Sciences University of Sydney NSW Australia; ^2^ 7800 School of Chemical Engineering University of New South Wales Sydney NSW Australia; ^3^ Sydney Informatics Hub University of Sydney Sydney NSW Australia; ^4^ 7799 Douglas Cohen Department of Paediatric Surgery Faculty of Medicine and Health The Children’s Hospital at Westmead Clinical School The University of Sydney School of Medicine Sydney NSW Australia; ^5^ School of Life and Environmental Sciences Charles Perkins Centre (D17) University of Sydney Sydney NSW 2006 Australia

## Abstract

The epidermis maintains a cellular calcium gradient that supports keratinocyte differentiation from its basal layers (low) to outer layers (high) leading to the development of the stratum corneum, which resists penetration of UV radiation. The calcium‐sensing receptor (CaSR) expressed in keratinocytes responds to the calcium gradient with signals that promote differentiation. In this study, we investigated whether the CaSR is involved more directly in protection from UV damage in studies of human keratinocytes in primary culture and in mouse skin studied *in vivo*. siRNA‐directed reductions in CaSR protein levels in human keratinocytes significantly reduced UV‐induced direct cyclobutane pyrimidine dimers (CPD) by ~80% and oxidative DNA damage (8‐OHdG) by ~65% compared with control transfected cells. Similarly, in untransfected cells, the CaSR negative modulator, NPS‐2143 (500 nm), reduced UV‐induced CPD and 8‐OHdG by ~70%. NPS‐2143 also enhanced DNA repair and reduced reactive oxygen species (ROS) by ~35% in UV‐exposed keratinocytes, consistent with reduced DNA damage after UV exposure. Topical application of NPS‐2143 also protected hairless Skh:hr1 mice from UV‐induced CPD, oxidative DNA damage and inflammation, similar to the reductions observed in response to the well‐known photoprotection agent 1,25(OH)_2_D_3_ (calcitriol). Thus, negative modulators of the CaSR offer a new approach to reducing UV‐induced skin damage.

## Graphical abstract

NPS‐2143, a CaSR antagonist, reduces both UV‐induced direct DNA damage, cyclobutane pyrimidine dimers (CPD), as well as oxidative DNA damage, 8‐hydroxy‐2′‐deoxyguanosine (8‐OHdG) through a combination of reduced ROS and enhanced DNA repair. It may work on either plasma membrane or endoplasmic reticulum (ER) localized CaSR.

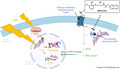

**Abbreviations**
1,25(OH)2D3: 1α,25‐dihydroxyvitamin D3
8‐OHdG: 8‐hydroxy‐2'‐deoxyguanosine
CaSR: calcium sensing receptor
CPD: cyclobutane pyrimidine dimer
ER: endoplasmic reticulum
ROS: reactive oxygen species
siRNA: small interfering RNA
ssUV: solar‐simulated UV irradiation
UDS: unscheduled DNA synthesis
VDR: vitamin D receptor

## INTRODUCTION

DNA damage is one of the key biological processes that contributes to UV‐induced skin carcinogenesis (1, 2). UVB (280–315 nm) directly excites the nucleobases in DNA and results in the oxygen‐independent formation of two dimeric photoproducts: cyclobutane pyrimidine dimers (CPDs) and (6‐4) pyrimidine‐pyrimidines. In keratinocytes, UV exposure also induces production of reactive oxygen species (ROS), including superoxide (O_2_^•−^), singlet oxygen (^1^O_2_), hydrogen peroxide (H_2_O_2_) and the hydroxyl free radical (^•^OH) (3, 4). Oxidative modifications to DNA, proteins, lipids and other cellular components occur when ROS levels exceed the capacity of intrinsic antioxidant defense mechanisms (5). Guanine is particularly susceptible to oxidative stress, forming 8‐hydroxy‐2′‐deoxyguanosine (8‐OHdG), due to its lower redox potential compared with the other three bases in DNA (6, 7, 8). Keratinocytes, like other mammalian cells, are equipped with DNA repair systems including base excision repair (BER), mismatch repair, double‐stranded break repair and nucleotide excision repair (NER) (9). Inadequate repair of UV‐induced DNA damage leads to genetic mutations and immune suppression, and in some cases progression to skin carcinogenesis (1, 10, 11, 12).

The epidermis maintains a cellular calcium gradient that increases from the inner basal and spinous layers to the outer stratum granulosum (13, 14). This gradient supports outwardly orientated keratinocyte differentiation and, thus, the development of the stratum corneum. The CaSR in keratinocytes reads and responds to the cellular gradient either intracellularly, in organelles such as the endoplasmic reticulum (ER) and/or Golgi (15, 16), where calcium ions are stored in millimolar concentration, or via its expression at the cell surface (13). Raising [Ca^2+^]_o_ above 0.1 mm, causes a transient increase in [Ca^2+^]_i_ through activation of the phospholipase C pathway leading to increased local levels of inositol 1,4,5 tris‐phosphate (IP_3_) and consequent release of Ca^2+^ from intracellular stores in the ER and Golgi (17, 18). Increased differentiation depends on a sustained phase of Ca^2+^ mobilization induced by elevated [Ca^2+^]_o_, which in turn seems to be due to increased Ca^2+^ influx through Ca^2+^ release‐activated Ca^2+^ entry mediated by the store‐operated channels. These are activated by the depletion of intracellular Ca^2+^ stores and activation of the IP_3_‐receptor (19). Although thickening of the stratum corneum in response to UV exposure, due to keratinocyte proliferation and differentiation, is a natural defense mechanism (20, 21), little is known about the possible role of intracellular or plasma membrane CaSR and its ligands in photobiology.

In contrast, it is well established that 1,25‐dihydroxyvitamin D_3_ [1,25(OH)_2_D_3_ or 1,25D], the hormonally active form of vitamin D, is photoprotective. 1,25(OH)_2_D_3_ reduces UV‐induced CPDs in human keratinocytes, melanocytes and fibroblast cultures, as well as in human skin *in vivo* and *ex vivo* (22, 23, 24, 25, 26). 1,25(OH)_2_D_3_ also decreases oxidative damage and 8‐OHdG formation (26, 27). When the vitamin D receptor (VDR) is knocked out, mice are more susceptible to chemical or UV‐induced skin tumors (28, 29) and mice in which there is tissue‐selective double knock‐out of the VDR and the calcium‐sensing receptor (CaSR) in the epidermis exhibit spontaneous development of squamous cell carcinomas (30, 31).

To investigate the role of the CaSR in the photo‐sensitivity of skin keratinocytes, we used siRNA knockdown or treatment with NPS‐2143, a negative modulator (calcilytic) of the CaSR (32). Calcilytics are well‐known systemically for inducing acute stimulation of parathyroid hormone (PTH) secretion, which is under tonic inhibitory control by the CaSR (32), but their effects on the skin, and on UV‐induced damage in particular, are unknown. Thus, we investigated the effects of CaSR knock‐down and treatment with NPS‐2143, on UV‐exposed keratinocytes to investigate the role of the CaSR in photo‐induced damage and its potential value as a target in photo‐protection.

## MATERIALS AND METHODS

Keratinocytes were cultured as previously described (23) from skin samples removed at elective surgery, with written informed consent from subjects or their parents/guardians, under University of Sydney Human Research Ethics Committee protocol no. 2015/063. Keratinocyte culture media was changed to DMEM without additives such as EGF and cholera toxin for 24 h before experiments to allow cells to become quiescent (33).

### DNA damage in vitro

Keratinocytes were seeded at a density of 20000 cells well^−1^ on 5 mm glass coverslips (Menzelgläser) in 96‐well plates and irradiated with an Oriel 1000W xenon‐arc lamp solar simulator (Newport Corporation). The spectral output of this lamp has been previously published (figure S6 in (34)). Irradiation used for this study was 400 mJ cm^−2^ UVB and 3600 mJ cm^−2^ UVA (4000 mJ cm^−2^), as measured by an OL754 radiometer (Optronics Laboratories Inc.). This dose of UV did not increase caspase activity or decrease total DNA content in keratinocytes for up to 6 h after exposure (34). Non‐irradiated control cells (SHAM‐vehicle) were located on the same plate as irradiated cells and treated in the same way as UV‐exposed cells but covered with foil to protect from ssUV. Immediately prior to UV irradiation, medium was replaced with irradiation buffer (phosphate‐buffered saline containing 10mM d‐glucose without phenol red). After UV, irradiation buffer was replaced with supplement‐free keratinocyte growth medium (KGM) containing following treatment as indicated, for 3 h: vehicle (0.1% v/v spectroscopic grade ethanol [Merck]), 1,25‐dihydroxyvitamin D_3_ [1,25(OH)_2_D_3_ or 1,25D; Sapphire Bioscience Pty Ltd.] at 1nM in ethanol, as the positive control (23, 24); CaCl_2_ or NPS‐2143 (HY‐1007 MCE^®^; Medchem Express), in ethanol. CPD, measured as thymine dimers, or 8‐OHdG were assayed by immunohistochemical staining and image analysis as previously described (27, 34). Staining and image analysis for CPD and 8‐OHdG produced similar results to those obtained using endonuclease detection of the lesion, followed by Comet assay (27, 35).

### DNA damage and skin edema in mice

The *in vivo* studies were approved by the Animal Ethics Committee of the University of Sydney (project no. 2015/794). Housing conditions and the solar simulator used for irradiation of mice have been described previously (24). Mice were subjected to a single exposure of 3 minimal erythemal doses of UV (UVB value at 399 mJ cm^−2^) (24, 36). Mice were treated topically over ~7 cm^2^ on the irradiated dorsal skin with 100 μL of the vehicle only—ethanol:propylene glycol:water 2:1:1 as previously described (24) or with 1,25(OH)_2_D_3_ or NPS‐2143 immediately after irradiation. The dose of NPS‐2143 was chosen as equivalent to 20X (228 pmol cm^−2^) or 200X (2280 pmol cm^−2^) that of an effective dose of 1,25(OH)_2_D_3_ 11.4 pmol cm^−2^. Biopsies (2 cm × 2 cm squared dorsal skin) were taken from UV‐irradiated, treatment applied dorsal skin 3 h post‐UV, paraffin‐embedded and processed for CPD as previously described (24). Non‐irradiated samples as SHAM control were obtained from the abdomen. Three areas of each section were analyzed (triplicates). Other mice, subjected to the same UV exposure and treatments had dorsal skin thickness measured with calipers as previously described (24) before exposure (day zero) and each day up to 7 days following UV.

### Small interfering RNA transfection of keratinocytes

Primary human keratinocytes were seeded as described above. For siRNA experiments, at 80% confluence, the culture medium was replaced with Opti‐MEM™ serum‐free medium (Thermofisher Scientific), 20 min prior to transfection. Transfection of siRNA was carried out according to the manufacturer’s instructions and as previously described (35). CaSR siRNA (siCaSR: sc‐44373), a pool of three target‐specific siRNAs (sequence in Table S1) and scrambled control siRNA (siCtrl: sc‐47741) (Santa Cruz). Briefly, siRNA at 25 nm were transfected into keratinocytes in the presence of 10% (v/v) lipid‐based transfection reagent (Santa Cruz), diluted in Opti‐MEM^TM^ overnight under normal growth conditions. The following day, an equal volume of 10% (v/v) fetal calf serum (FCS) in supplement‐free KGM (23) was added to each well to give 5% FCS overall. A further 24–48 h period of culture was permitted to achieve sufficient protein knockdown. This was verified by Western Blot and densitometry analysis with ImageJ (National Institute of Health) (35). siRNA transfection of keratinocytes was performed 48 h before UV exposure or treatments.

### Western blot

Keratinocytes were plated directly in 6‐well plates in KGM with supplements. Following the indicated treatments, cells were lysed and subjected to western blot as previously described (37) with lysis buffer prepared from 62.5 mm Tris–HCl pH 6.8, 50 mm DTT, 2% (w/v)SDS, 10% (v/v) glycerol, 0.01% bromophenol blue as color indicator, and with protease inhibitor cocktail (10 mm 4‐benzenesulfonyl fluoride hydrochloride, 10 μm pepstatin A, 50 μm bestatin‐HCl, 2.5 mm sodium fluoride, 1 mm sodium orthovanadate, 3 mm sodium pyrophosphoate and 3 mm β‐glycerophosphate) added freshly on the day. Lysates were then sonicated to reduce viscosity, reduced with 2% (v/v) β‐mercaptoethanol (ICN Biomedicals) and thermally denatured at 70°C for 5 min. After centrifugation and cooling to RT, 20 μL lysates were loaded into pre‐prepared SDS polyacrylamide gels (10% resolving/4% stacking), with electrophoresis carried out at 120 V for 90 min. Transfer of protein to nitrocellulose membrane (Amersham™ Protran^®^ 0.45 μm NC; GE Healthcare) was achieved at 25 V overnight at 4°C. The membrane was then blocked with 5% BSA (w/v in Tris‐Buffered Saline with Tween [TBS‐T], pH7.2) for 1 h before being incubated with primary antibody: anti‐CaSR at 1 µg mL^−1^ (HL1499; Sigma‐Aldrich), overnight at 4°C or anti‐tubulin at 1 μg mL^−1^ (mouse monoclonal, SC‐5286; Santa Cruz). The next day, the membrane was washed with TBS‐T three times and incubated with either HRP‐linked goat anti‐rabbit (Santa Cruz) or goat anti‐mouse secondary antibody (Cell Signaling Technology), as appropriate, for 1h at room temperature. The membrane was again washed three times with TBS‐T before adding chemiluminescence substrate 1:1 (Merck) for band detection. The band was imaged with ChemiDoc™ imaging system (Bio‐Rad Laboratories, Inc.) at the Bosch Research Institute (University of Sydney) and densitometry was carried out using Image J.

### Unscheduled DNA synthesis

Incorporation of 5‐ethynyl‐2′‐deoxyuridine (EdU) into nuclear DNA was detected using iClick™ EdU Alexa Fluor 488 Imaging Kit (Catalogue number A003) according to the manufacturer’s instructions (Applied BioProbes), as previously described (34). Incorporation of this thymidine analog in a punctate pattern, in keratinocytes cultured without growth factors, measures unscheduled DNA synthesis rather than DNA replication (38). Visualization of nuclear EdU‐incorporation was carried out by confocal laser microscopy (LSM 510 Meta; Zeiss, Oberkochen Germany). Densitometry was performed using Image J software (National Institute of Health, Maryland). Stained cells were manually counted from the images taken by Zeiss LSM 510 Meta confocal microscope. Fluorescence intensity was measured in cells that were above the detection threshold and considered "UDS positive."

### ROS measurement

ROS levels were determined using the ROS‐Glo™ H_2_O_2_ Assay (Promega, WI) according to the manufacturer's instructions, as described previously (34).

### Statistical analyses

The results are based on triplicates of each treatment with keratinocytes from different donors in each experiment, unless otherwise stated. Data were normalized to SHAM of each experiment and pooled from three or more experiments, expressed as mean ± SEM, unless otherwise indicated. All statistical analyses were performed using the GraphPad Prism version 8.0 statistical program (GraphPad Software Inc.). Unless otherwise stated, the analysis of comparisons between treatment groups was made using Mixed Effects Model analysis with Sidak’s post‐comparison test.

## RESULTS

### siRNA‐directed knockdown of CaSR reduced UV‐induced DNA damage in primary human keratinocytes

**Figure 1:**
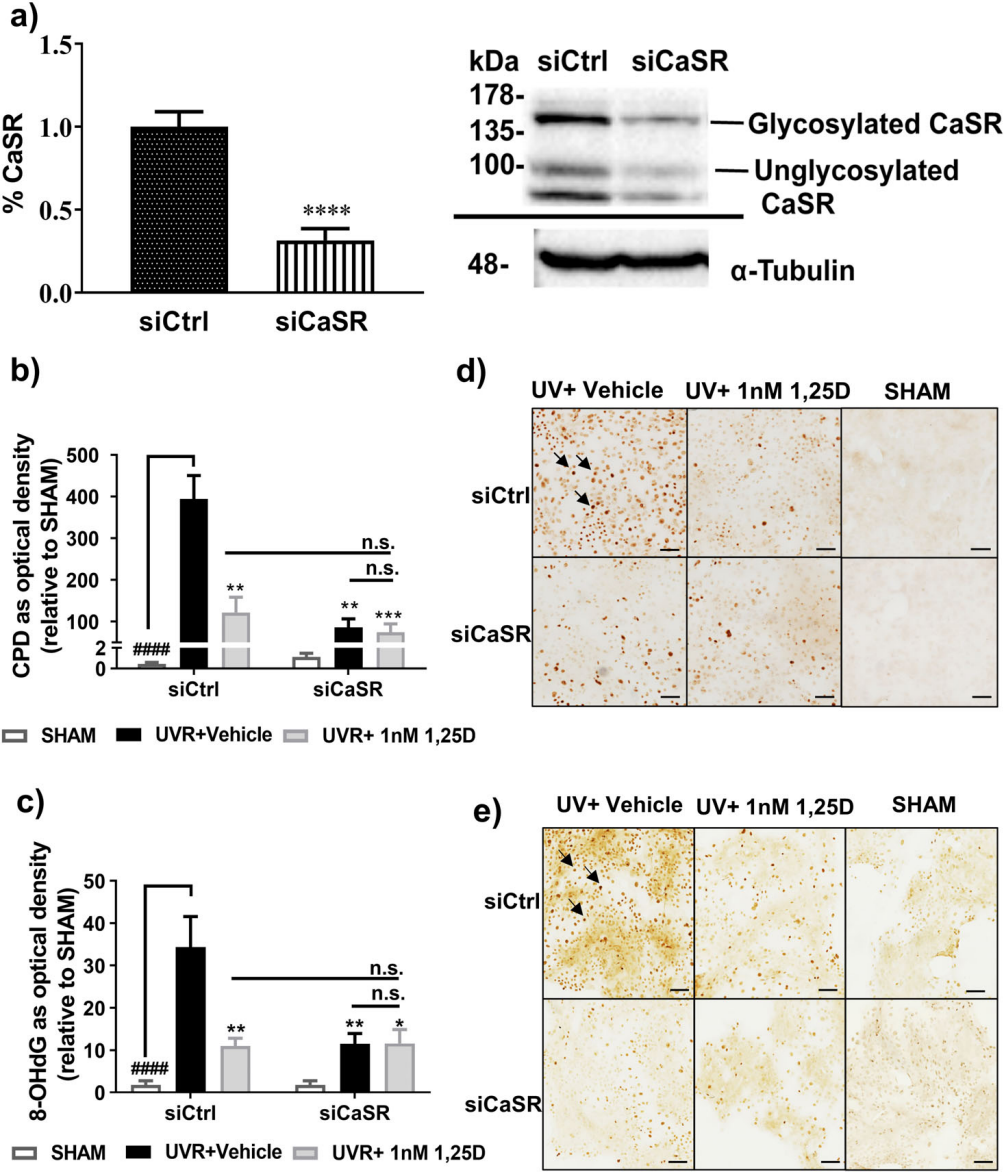
siRNA‐mediated reduction in CaSR protein levels reduced UV‐induced DNA damage in primary human keratinocytes. (a) Western blot image and densitometry of CaSR densities 48 h post siRNA transfection, at the time of UVR exposure. Expression was normalized to siCtrl to pool data from three independent experiments. α‐Tubulin was a loading control. ****P < 0.001 when compared with CaSR protein level under siCtrl condition. DNA damage (b) CPD or (c) 8‐OHdG (*y*‐axis) by immunohistochemistry and image analysis in either siCtrl or siCaSR transfected kertainocytes at 3 h post‐UV with or without 1,25(OH)_2_D_3_ (1,25D). Data were shown as mean ± SEM (*n* = 9) relative to SHAM (non‐irradiated). ***P < 0.001, **P < 0.01 and *P < 0.05, when compared with siCtrl UV + vehicle; n.s., not significant among treatments. ^####^ *P* < 0.0001 significantly different from UV + vehicle. Photomicrographs of (d) CPD and (e) 8‐OHdG in the presence of siCtrl or siCaSR. Black arrows point to the dark brown staining in nuclei indicating the presence of CPD or 8‐OHdG. Scale bar = 100 μm.

Human primary keratinocytes were transfected with small interfering RNA (siRNA) targeting CaSR (siCaSR) or non‐specific siRNA (siCtrl). siCaSR transfection reduced CaSR protein to ~40% (*P* < 0.0001) of the control transfected cells (Fig. 1a). In the presence of siCtrl, UV induced substantial increases in CPD levels by ~500‐fold relative to non‐irradiated cells (siCtrl SHAM) (Fig. 1b,d) and a well‐recognized photoprotective agent, 1 nm 1,25(OH)_2_D_3_ (22, 23, 26) added immediately after UV exposure, suppressed UV‐induced CPD levels by ~75% (*P* < 0.01) (Fig. 1b,d). In siCaSR‐transfected keratinocytes exposed to UV, CPD levels were significantly reduced in both the absence and presence of 1,25(OH)_2_D_3_ to levels observed in non‐irradiated cells (siCaSR SHAM) (*P* < 0.01, Fig. 1b,d). A similar pattern of reduction was observed in UV‐induced oxidative DNA damage as measured by 8‐OHdG in response to the siCaSR condition (Fig. 1c,e).

### NPS‐2143 protected keratinocytes against UV‐induced DNA damage

**Figure 2:**
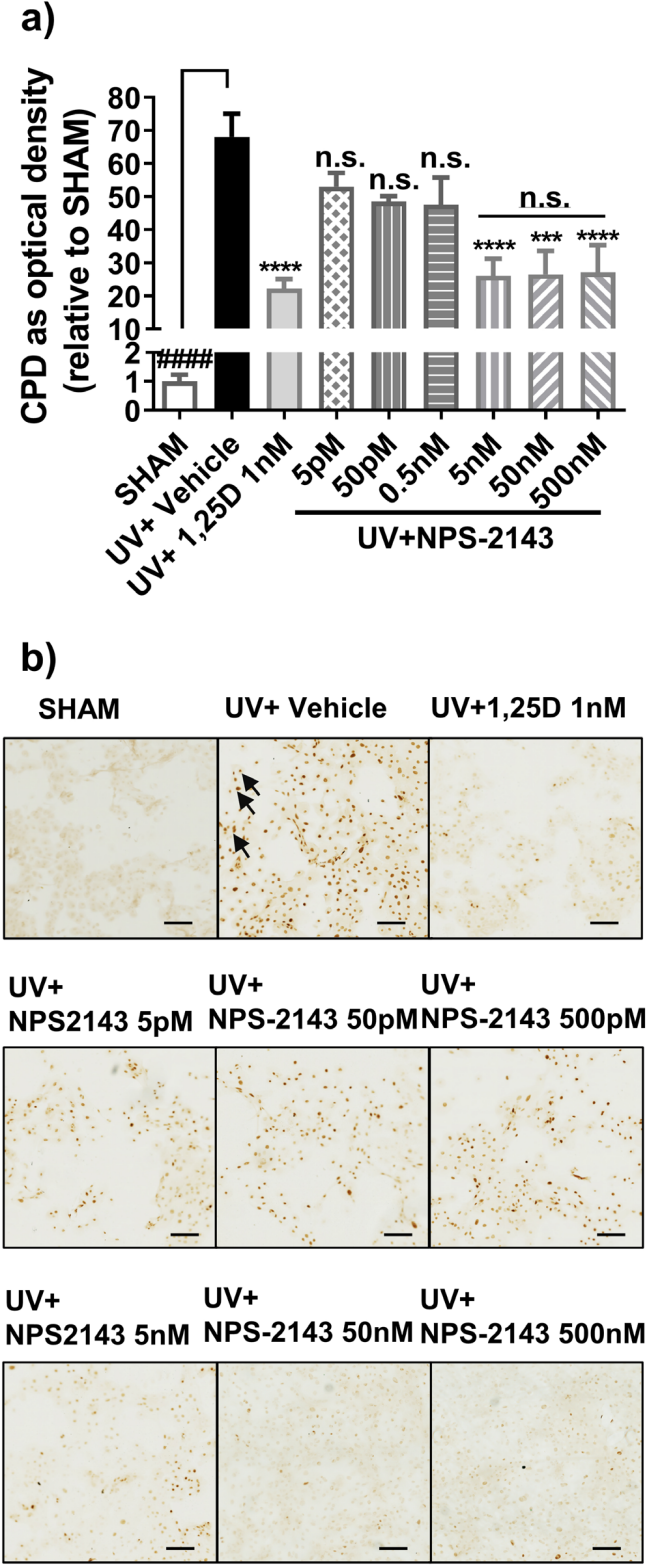
Suppression of UV‐induced CPD by NPS‐2143 in human keratinocytes. (a) CPD *in vitro* by immunohistochemistry and image analysis at 3 h post UV with treatments as indicated. Media contained 1.0 mm Ca^2+^_o_. Data were shown as mean + SEM (*n* = 9). ****P < 0.0001, ***P < 0.001, n.s. not significant, when compared with UV+vehicle. n.s. (with bar) not significantly different from one another. ####, *P* < 0.0001 significantly different from UV+vehicle b) Photomicrographs of CPD in vitro b). Black arrows point to the dark brown staining in nuclei indicating the presence of CPD. Scale bar = 100 µm

We then tested the effects of various concentrations of NPS‐2143 on UV‐induced CPD levels in keratinocytes. NPS‐2143 was used in the range 5 pm to 500 nm. This concentration range was chosen based on previous results from studies in HEK‐293 cells, which showed an antagonistic NPS‐2143 response (32). In our primary keratinocytes, NPS‐2143 had no effect in the range 5 pm–0.5 nm but suppressed UV‐induced CPD by ~50% at all concentrations tested in the range 5 nm to 500 nm (*P* < 0.001, Fig. 2). From these results, a fixed NPS‐2143 concentration of 500 nm was chosen for subsequent experiments.

**Figure 3:**
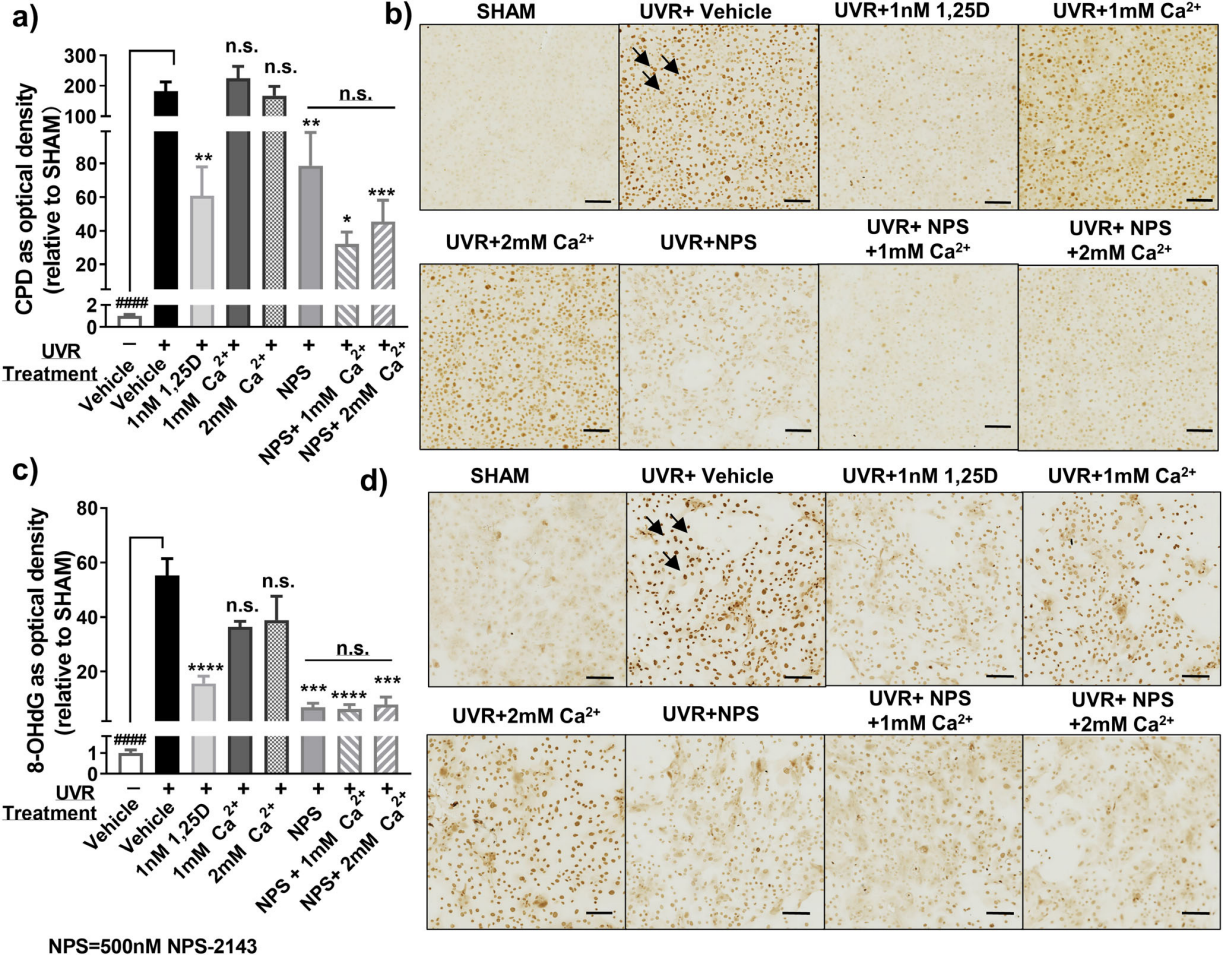
NPS‐2143 protects human primary keratinocytes from UV‐induced DNA damage at both 1.0 and 2.0 mm Ca^2+^_o_. (a) CPD and (c) 8‐OHdG by immunohistochemistry and image analysis at 3 h post UV with treatments as indicated. NPS‐2143 concentration was 500 nm and that of 1,25(OH)_2_D_3_ was 1 nm. Data were shown as mean ± SEM (*n* = 9). ****P < 0.0001, ***P < 0.001, **P < 0.01 and *P < 0.05, n.s., not significant when compared with UV + vehicle. n.s. (with bar) not significantly different from one another. ####, *P* < 0.0001 significantly different from UV + vehicle. Photomicrographs of (b) CPD and (d) 8‐OHdG. Black arrows point to the dark brown staining in nuclei indicating the presence of CPD or 8‐OHdG. Scale bar = 100 μm.

We next tested the effect of extracellular calcium (Ca^2+^_o_) concentration on UV‐induced DNA damage and its sensitivity to NPS‐2143. Raising Ca^2+^_o_ from its baseline level of 0.16 mm to 1.0 mm or 2.0 mm had no effect on the UV‐induced increase in CPD or 8‐OHdG levels in human keratinocytes (Fig. 3). NPS‐2143 (500 nm) significantly reduced UV‐induced CPD (*P* < 0.01) and 8‐OHdG (*P* < 0.001) levels in the presence of all three Ca^2+^_o_ concentrations tested (Fig. 3).

### NPS‐2143 enhanced DNA repair after UV

**Figure 4:**
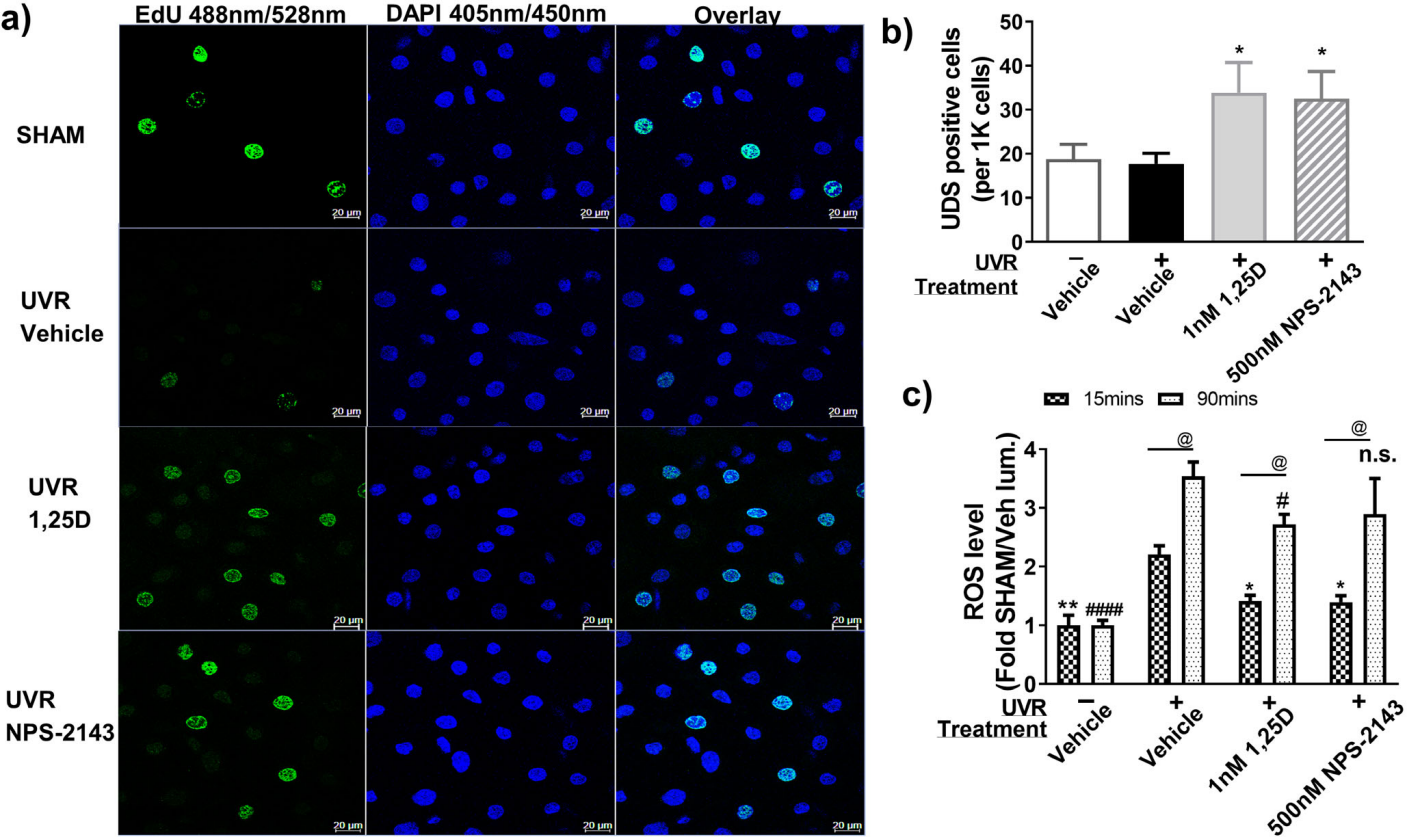
NPS‐2143 enhanced repair and reduced ROS in UV‐exposed keratinocytes. (a) Confocal images of thymidine analog EdU after 90 min with or without treatments in the nucleus of keratinocytes (DAPI counterstained). EdU positive cells were shown with green punctate staining, scale bar = 20 µm. UDS data were presented as (b) Numbers of EDU positive cells per 1000 cells counted, mean ± SEM. (c) ROS levels 15 min (dark, crosshatched bars) or 90 min (dotted, clear bars) after irradiation, measured in luminescence units normalized to SHAM (Mean ± SEM *n* = 6). **P < 0.01 and *P < 0.05 compared with UV + vehicle at 15 min, ^#^ *P* < 0.05, ^####^ *P* < 0.0001 or n.s. when compared with UV + vehicle at 90 min; @*P* < 0.01 when compared with the same treatment group at different time points.

We investigated whether NPS‐2143 promoted DNA repair in the context of UV‐induced damage using the unscheduled DNA synthesis (UDS) assay (39), which measures incorporation of the fluorescent thymidine analog 5‐ethynyl‐2′‐deoxyuridine (EdU) into DNA. In these quiescent cells, a punctate pattern of staining was observed, indicative of DNA repair (38). Ninety minutes after UV exposure, staining remained low in vehicle‐treated keratinocytes. Treatment with either 1,25(OH)_2_D_3_ (1 nm) or NPS‐2143 (500 nm) increased the proportion of UDS positive cells (*P* < 0.05 for both) (Fig. 4a,b) and the average intensity of fluorescence per positive cell (*P* < 0.05 for both) (Figure S1) at this time after UV exposure.

### NPS‐2143 reduced UV‐induced oxidative stress

ROS were then measured in keratinocytes after UV challenge, at a dose which induces oxidative DNA damage (4). Test treatments were again added immediately after UV exposure, as in previous experiments. At 15 min post‐UV irradiation, ROS levels were ~35% lower in cells treated with 1 nm 1,25(OH)_2_D_3_ (*P* < 0.05) or with 500 nm NPS‐2143 (*P* < 0.05) when compared with vehicle‐treated cells (Fig. 4c). At 90 min post‐irradiation, however, whereas 1,25(OH)_2_D_3_ continued to significantly suppress ROS levels by 25%, ROS levels in cells treated with NPS‐2143 were not significantly different from either cells exposed to the vehicle control or to 1,25(OH)_2_D_3_ at that time‐point (Fig. 4c).

### NPS‐2143 protected mouse skin against UV‐induced DNA damage and edema

**Figure 5:**
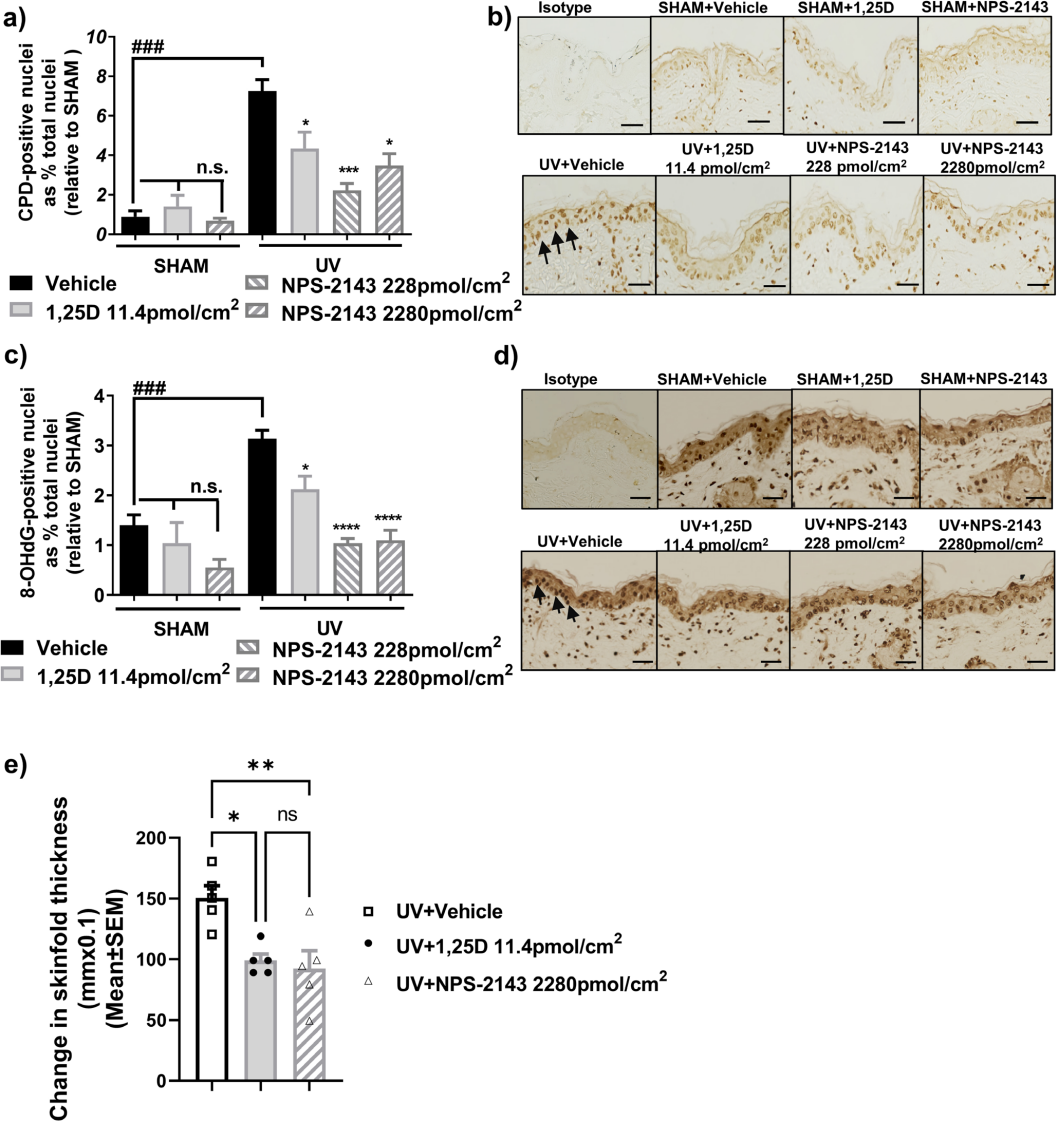
NPS‐2143 inhibits UV‐induced DNA damage and skin edema in mice following acute UV irradiation. Mice were exposed to 3 MED of ssUV and immediately treated on the UV‐irradiated dorsum with vehicle, 1,25(OH)_2_D_3_ or NPS‐2143 as indicated. (a) CPD or (c) 8‐OHdG by immunohistochemistry and image analysis in female SKH:hr1 mice, at 3 h post‐UV exposure, three mice per group, with similar results obtained in a second set of mice. ****P < 0.0001, ***P < 0.001, *P < 0.05, compared with UV + vehicle. n.s. (with bar) not significantly different from one another. ####, *P* < 0.0001 significantly different from UV + vehicle. Photomicrographs of CPD (b) or 8‐OHdG (d) in mouse skin. Black arrows point to the dark brown staining in nuclei indicating the presence of CPD or 8‐OHdG. Scale bar = 100 μm. (e) Skin edema was measured as skinfold thickness on the 4^th^ day after UVR exposure. Data were expressed as mean change in dorsal skinfold thickness + SEM compared with non‐irradiated skinfold thickness. **P < 0.01 and *P < 0.05 when compared with UV + vehicle group. *n* = 5 mice per group.

Although keratinocytes in primary cell culture provide a powerful approach for studying epidermal biology, they imperfectly model the various cell types and three‐dimensional topology of the epidermis (40). NPS‐2143, painted topically on Skh:hr1 mice immediately after exposure to 3 minimal erythema doses (3MED) of ssUV at two doses (228 pmol cm^−2^ and 2280 pmol cm^−2^), caused significant reductions in the levels of both CPD and 8‐OHdG (*P* < 0.001), in a manner similar to 1,25(OH)_2_D_3_ (Fig. 5a–d). Skin edema is an inflammatory response to UV irradiation. We observed that skinfold thickness increased daily reaching a maximum at day 4 post‐ssUV, at approximately twice the normal skinfold thickness. It then decreased gradually towards baseline. As shown in Fig. 5e, on day 4 after UV exposure, dorsal skin thickness was significantly reduced by treatment with NPS‐2143 (*P* < 0.01) or 1,25(OH)_2_D_3_ (*P* < 0.01).

## DISCUSSION

Although the CaSR is required for keratinocyte differentiation and barrier formation in skin (13, 17, 41), its significance for the modulation of DNA damage and repair after UV exposure is unknown. The results presented in this study provide evidence that the CaSR protein itself modulates UV‐induced DNA damage and repair in skin. Thus keratinocytes in which (1) the level of CaSR protein has been reduced prior to UV exposure; or (2) treated with its negative modulator NPS‐2143 shortly after UV exposure, exhibited significantly attenuated UV‐induced increases in CPD and 8‐OHdG. The results demonstrate that NPS‐2143 was maximally effective in reducing UV‐induced DNA damage at a concentration of 50–500 nm, consistent with its stimulatory impact on PTH in other cell types (32).

### What might contribute to reduced DNA damage in the presence of NPS‐2143?

CPD generation predominantly arises from direct absorption of UV by thymidine bases in DNA (42). 8‐OHdG generation arises from oxidative damage to deoxyguanosines in DNA from excess ROS released by UV‐damaged mitochondria (4, 43). Increased repair of both CPD and 8‐OHdG is critical (44, 45). Under the conditions of this experiment, where the keratinocytes are not proliferating in the absence of growth factors, unscheduled DNA synthesis can be used to quantify DNA repair (39). NPS‐2143, like 1,25(OH)_2_D_3_ (34), enhanced DNA repair, evidenced by increased incorporation of fluorescence‐labeled thymidine analog, 5‐ethynyl‐2′‐deoxyuridine (Fig. 4, Figure S1). NPS‐2143 has been reported to inhibit epidermal would healing in mice (46), consistent with an inhibitory effect on keratinocyte proliferation. Reduced rates of proliferation associated with arrest in G2, provide increased time for DNA repair (47). NPS‐2143 has also been reported to suppress the proliferation of other cell types, such as colon cancer cells (48).

NPS‐2143 may reduce UV‐induced DNA damage via suppression of ROS production. In this study, keratinocytes treated immediately after exposure to UV with either 1,25(OH)_2_D_3_ or NPS‐2143 exhibited significantly lower levels of ROS (Fig. 4c). Excess intracellular ROS, arising from UV‐exposed mitochondria (4, 49), directly promotes the formation of 8‐OHdG (50). Excess ROS has also been reported to impair DNA repair by damaging DNA repair proteins (51). We were unable to detect a change in the level of the key DNA repair protein, xeroderma pigmentosum, complementation group C, also known as XPC, by western blotting in UV‐exposed keratinocytes treated with NPS‐2143 (data not shown). Recent evidence suggests that rates of dissociation of XPC from damaged DNA, rather than the total amount of XPC, determines the rate of DNA repair following UV exposure (52). NPS‐2143 treatment or CaSR knockdown have also been reported to reduce ROS production stimulated by exposure to melamine in kidney tubular cells (53).

Significant reductions in UV‐induced CPD and 8‐OHdG were also observed in this study after treatment with topical NPS‐2143 in mice, indicating that it is effective on UV‐exposed native skin *in vivo* as well as on UV‐exposed keratinocytes *in vitro*. The protection by NPS‐2143 against UV‐induced DNA damage *in vivo* was similar in magnitude to that produced by the well‐described photoprotective agent, 1,25(OH)_2_D_3_ (22, 23, 26). In these mice, NPS‐2143 also reduced UV‐induced inflammation (Fig. 5e). A consistent inhibitory effect of NPS‐2143 on inflammation in several other systems has been reported, with reported mechanisms that include reduced activation of the NLRP inflammasome (54, 55, 56) and reductions in the pro‐inflammatory cytokine IL‐6 (57, 58, 59, 60).

In mouse studies, which examined effects of CaSR knockdown (13, 17, 41), 90% knockdown of the CaSR protein in the epidermis eventually led to disruption of the epidermal permeability barrier. Treatment of immortalized keratinocytes with NPS‐2143 has also been reported to inhibit some measures of cellular differentiation (61). In this study, siRNA knockdown of CaSR in keratinocytes was only 60%. The inhibition of the CaSR by treatment with NPS‐2143 enhanced keratinocyte survival after UV, as measured by cell titer blue, in a similar manner to 1,25(OH)_2_D_3_ (Figure S2). Furthermore, *in vivo* functional outcomes after treatment with NPS‐2143 were similar to those seen with the known photoprotective agent, 1,25(OH)_2_D_3_. This indicates that even if some epithelial function was altered after application of topical NPS‐2143 to mice, it did not prevent the significant reductions in UV‐induced CPD or oxidative DNA damage or in skin edema, a marker of inflammation, observed with this agent.

At first sight, the findings of photoprotective effects of CaSR knockdown or pharmacologic inhibition in human keratinocytes are inconsistent with reports that mice with epidermal double knockout of the VDR and CaSR spontaneously develop squamous cell carcinomas (30, 31). These tumors developed in the context of low calcium diets, however, and were not UV associated (30, 31). Studies of UV‐induced DNA damage in mice with single or double epidermal knockout of the VDR and/or CaSR may help to identify the mechanisms.

### Is intracellular CaSR involved?

UV exposure results in increased keratinocyte proliferation and differentiation leading to increased production of the protective stratum corneum at the surface of the skin (62). However, it is not clear which components of the UV‐induced molecular response including: (1) the production of CPD, ROS and 8‐OHdG; (2) local activation of 1,25OH_2_D_3_ synthesis and possibly; (3) local activation of the intracellular or plasma membrane CaSR are required for keratinocyte proliferation and differentiation.

The CaSR is synthesized in the ER and its N‐terminus is delivered into the ER lumen where it is subjected to co‐translational glycosylation and quality control checkpoints leading to its release and trafficking to the plasma membrane (63). Many activities of the CaSR are dependent on its expression at the plasma membrane, where it is activated by Ca^2+^_o_ and other nutrients, including L‐amino acids, and modulators including the polyamine spermine (64, 65) and inhibited by polyvalent anions including inorganic phosphate (66). CaSR expression has also been demonstrated in pre‐plasma membrane compartments in various cell types, including keratinocytes, revealing the existence of an intracellular reservoir of CaSR (18, 48, 67, 68, 69, 70). Previously it was assumed that the intracellular CaSR is inactive and held in storage for transit to the plasma membrane. More recently, immature ER‐localized CaSR has been reported to mediate a range of cellular actions (18, 48, 71) including in keratinocytes (18).

NPS‐2143 is an uncharged aromatic compound that is lipophilic and thus expected to penetrate intracellularly (71, 72) to impact the function of both plasma membrane and intracellularly located CaSR. In an extension of these studies, we used tunicamycin to prevent N‐linked glycosylation of the CaSR in primary human keratinocytes and thus impair trafficking to the plasma membrane. Consistent with this idea, tunicamycin markedly suppressed CaSR expression in the plasma membrane (Figure S3). Interestingly, tunicamycin had no effect on NPS‐2143 dependent suppression of UV‐induced 8‐OHdG but almost abolished NPS‐2143‐dependent reductions in UV‐induced CPD (Figure S4a–d). These results suggest that UV‐induced 8‐OHdG protection is dependent on an intracellular pool of the CaSR, whereas UV‐induced CPD protection requires CaSR expressed in the plasma membrane. Also consistent with this idea was the lack of effect of raised medium Ca^2+^ concentration (to 2mM) on DNA damage in the either the absence or presence of NPS‐2143.

In conclusion, CaSR knockdown or exposure to the CaSR inhibitor NPS‐2143 protected human keratinocytes and native mouse skin against UV‐induced DNA damage including the direct production of CPD and ROS‐dependent production of 8‐OHdG to a similar extent to that provided by 1,25(OH)_2_D_3_. The photoprotective activity of NPS‐2143 arose at least in part from enhanced DNA repair and suppressed production of ROS. Regarding the mechanisms by which the CaSR modulates UV‐induced skin damage, studies with tunicamycin support the proposal that both plasma membrane and intracellular pools of CaSR mediate the suppressive effects of its negative modulator NPS‐2143. Further investigation is required to identify the specific signaling mechanisms that support these two distinct processes. Importantly, this study identifies the CaSR as a novel target in strategies aimed at protecting the skin against UV‐induced DNA damage.

## CONFLICTS OF INTEREST

The authors state that they have no conflicts.

## Supporting information


**Figure S1**. NPS‐2143 promoted repair.
**Figure S2**. NPS‐2143 and 1,25(OH)_2_D_3_ enhanced cell survival after UV.
**Figure S3**. Tunicamycin reduced expression of CaSR at the plasma membrane.
**Figure S4**. Tunicamycin pre‐treatment had no effect on NPS‐2143 suppression of UV‐induced 8‐OHdG.
**Table S1**. siRNA duplex sequences obtained from Santa Cruz Biotechnology.

## References

[1] Kripke, M. L. , P. A. Cox , L. G. Alas and D. B. Yarosh (1992) Pyrimidine dimers in DNA initiate systemic immunosuppression in UV‐irradiated mice. Proc. Natl Acad. Sci. 89, 7516–7520. 1502162 10.1073/pnas.89.16.7516 PMC49741

[2] Nakagawa, A. , N. Kobayashi , T. Muramatsu , Y. Yamashina , T. Shirai , M. W. Hashimoto , M. Ikenaga and T. Mori (1998) Three‐dimensional visualization of ultraviolet‐induced DNA damage and its repair in human cell nuclei. J. Invest. Dermatol. 110, 143–148. 9457909 10.1046/j.1523-1747.1998.00100.x

[3] Heck, D. E. , A. M. Vetrano , T. M. Mariano and J. D. Laskin (2003) UVB light stimulates production of reactive oxygen species: Unexpected role for catalase. J. Biol. Chem. 278, 22432–22436. 12730222 10.1074/jbc.C300048200

[4] Aitken, G. R. , J. R. Henderson , S. C. Chang , C. J. McNeil and M. A. Birch‐Machin (2007) Direct monitoring of UV‐induced free radical generation in HaCaT keratinocytes. Clin Exp Dermatol 32, 722–727. 17953641 10.1111/j.1365-2230.2007.02474.x

[5] Pattison, D. I. and M. J. Davies (2006) Actions of ultraviolet light on cellular structures. EXS, 131–157. 16383017 10.1007/3-7643-7378-4_6

[6] Peak, M. J. , J. G. Peak and B. A. Carnes (1987) Induction of direct and indirect single‐strand breaks in human cell DNA by far‐ and near‐ultraviolet radiations: action spectrum and mechanisms. Photochem. Photobiol. 45, 381–387. 3562593 10.1111/j.1751-1097.1987.tb05390.x

[7] Peak, M. J. , J. G. Peak and C. A. Jones (1985) Different (direct and indirect) mechanisms for the induction of DNA‐protein crosslinks in human cells by far‐ and near‐ultraviolet radiations (290 and 405 nm). Photochem. Photobiol. 42, 141–146. 4048296 10.1111/j.1751-1097.1985.tb01552.x

[8] Cadet, J. , A. Grand and T. Douki (2014) Solar UV radiation‐induced dna bipyrimidine photoproducts: formation and mechanistic insights. In Photoinduced Phenomena in Nucleic Acids II, (Edited by M. Barbatti , Borin A. and Ullrich S. ), Vol 356, pp. 249–275. Topics in Current Chemistry, Springer, Cham, Cham, Germany. 10.1007/128_2014_553 25370518

[9] Matsumura, Y. and H. N. Ananthaswamy (2002) Short‐term and long‐term cellular and molecular events following UV irradiation of skin: Implications for molecular medicine. Expert Rev. Mol. Med. 4, 1–22. 10.1017/S146239940200532X 14585163

[10] Kripke, M. L. and M. S. Fisher (1976) Immunologic parameters of ultraviolet carcinogenesis. J. Natl Cancer Inst. 57, 211–215. 1003502 10.1093/jnci/57.1.211

[11] Applegate, L. A. , R. D. Ley , J. Alcalay and M. L. Kripke (1989) Identification of the molecular target for the suppression of contact hypersensitivity by ultraviolet radiation. J. Exp. Med. 170, 1117–1131. 2529340 10.1084/jem.170.4.1117 PMC2189477

[12] Halliday, G. M. (2005) Inflammation, gene mutation and photoimmunosuppression in response to UVR‐induced oxidative damage contributes to photocarcinogenesis. Mutat. Res. 571, 107–120. 15748642 10.1016/j.mrfmmm.2004.09.013

[13] Tu, C. L. , D. A. Crumrine , M. Q. Man , W. Chang , H. Elalieh , M. You , P. M. Elias and D. D. Bikle (2012) Ablation of the calcium‐sensing receptor in keratinocytes impairs epidermal differentiation and barrier function. J. Invest. Dermatol. 132, 2350–2359. 22622426 10.1038/jid.2012.159 PMC3434298

[14] Lee, S. E. and S. H. Lee (2018) Skin barrier and calcium. Annal. Dermatol. 30, 265–275. 10.5021/ad.2018.30.3.265 PMC5929942 29853739

[15] Leach, K. , F. M. Hannan , T. M. Josephs , A. N. Keller , T. C. Moller , D. T. Ward , E. Kallay , R. S. Mason , R. V. Thakker , D. Riccardi , A. D. Conigrave and H. Brauner‐Osborne (2020) International union of basic and clinical pharmacology. CVIII. Calcium‐sensing receptor nomenclature, pharmacology, and function. Pharmacol. Rev. 72, 558–604. 32467152 10.1124/pr.119.018531 PMC7116503

[16] Komuves, L. , Y. Oda , C. L. Tu , W. H. Chang , C. L. Ho‐Pao , T. Mauro and D. D. Bikle (2002) Epidermal expression of the full‐length extracellular calcium‐sensing receptor is required for normal keratinocyte differentiation. J. Cell Physiol. 192, 45–54. 12115735 10.1002/jcp.10107

[17] Tu, C. L. and D. D. Bikle (2013) Role of the calcium‐sensing receptor in calcium regulation of epidermal differentiation and function. Best Pract. Res. Clin. Endocrinol. Metab. 27, 415–427. 23856269 10.1016/j.beem.2013.03.002 PMC3713412

[18] Tu, C. L. , W. Chang and D. D. Bikle (2007) The role of the calcium sensing receptor in regulating intracellular calcium handling in human epidermal keratinocytes. J. Invest. Dermatol. 127, 1074–1083. 17124506 10.1038/sj.jid.5700633

[19] Putney, J. W. Jr (2005) Capacitative calcium entry: Sensing the calcium stores. J. Cell Biol. 169, 381–382. 15866892 10.1083/jcb.200503161 PMC2171919

[20] Brenner, M. and V. J. Hearing (2008) The protective role of melanin against UV damage in human skin. Photochem. Photobiol. 84, 539–549. 18435612 10.1111/j.1751-1097.2007.00226.x PMC2671032

[21] Scott, T. L. , P. A. Christian , M. V. Kesler , K. M. Donohue , B. Shelton , K. Wakamatsu , S. Ito and J. D'Orazio (2012) Pigment‐independent cAMP‐mediated epidermal thickening protects against cutaneous UV injury by keratinocyte proliferation. Exp. Dermatol. 21, 771–777. 23078399 10.1111/exd.12012 PMC3481176

[22] Dixon, K. M. , S. S. Deo , G. Wong , M. Slater , A. W. Norman , J. E. Bishop , G. H. Posner , S. Ishizuka , G. M. Halliday , V. E. Reeve and R. S. Mason (2005) Skin cancer prevention: A possible role of 1,25dihydroxyvitamin D3 and its analogs. J. Steroid. Biochem. 97, 137–143. 10.1016/j.jsbmb.2005.06.006 16039116

[23] Gupta, R. , K. M. Dixon , S. S. Deo , C. J. Holliday , M. Slater , G. M. Halliday , V. E. Reeve and R. S. Mason (2007) Photoprotection by 1,25 dihydroxyvitamin D3 is associated with an increase in p53 and a decrease in nitric oxide products. J. Invest. Dermatol. 127, 707–715. 17170736 10.1038/sj.jid.5700597

[24] Dixon, K. M. , A. W. Norman , V. B. Sequeira , R. Mohan , M. S. Rybchyn , V. E. Reeve , G. M. Halliday and R. S. Mason (2011) 1 alpha,25(OH)(2)‐vitamin D and a nongenomic vitamin D analogue inhibit ultraviolet radiation‐induced skin carcinogenesis. Cancer Prev. Res. 4 (9):1485–1494. 10.1158/1940-6207.CAPR-11-0165 21733837

[25] Slominski, A. T. , T. K. Kim , H. Z. Shehabi , I. Semak , E. K. Tang , M. N. Nguyen , H. A. Benson , E. Korik , Z. Janjetovic , J. Chen , C. R. Yates , A. Postlethwaite , W. Li and R. C. Tuckey (2012) In vivo evidence for a novel pathway of vitamin D(3) metabolism initiated by P450scc and modified by CYP27B1. FASEB J. 26 (9):3901–3915. 22683847 10.1096/fj.12-208975 PMC3425822

[26] Song, E. J. , C. Gordon‐Thomson , L. Cole , H. Stern , G. M. Halliday , D. L. Damian , V. E. Reeve and R. S. Mason (2013) 1alpha,25‐Dihydroxyvitamin D3 reduces several types of UV‐induced DNA damage and contributes to photoprotection. J. Steroid Biochem. Molec. Biol. 136, 131–138. 23165145 10.1016/j.jsbmb.2012.11.003

[27] Gordon‐Thomson, C. , R. Gupta , W. Tongkao‐on , A. Ryan , G. M. Halliday and R. S. Mason (2012) 1alpha,25 dihydroxyvitamin D3 enhances cellular defences against UV‐induced oxidative and other forms of DNA damage in skin. Photochem. Photobiol. Sci. 11, 1837–1847. 23069805 10.1039/c2pp25202c

[28] Ellison, T. I. , M. K. Smith , A. C. Gilliam and P. N. MacDonald (2008) Inactivation of the vitamin D receptor enhances susceptibility of murine skin to UV‐induced tumorigenesis. J. Invest. Dermatol. 128, 2508–2517. 18509362 10.1038/jid.2008.131 PMC4127033

[29] Teichert, A. E. , H. Elalieh , P. M. Elias , J. Welsh and D. D. Bikle (2011) Overexpression of hedgehog signaling is associated with epidermal tumor formation in vitamin D receptor‐null mice. J. Invest. Dermatol. 131, 2289–2297. 21814234 10.1038/jid.2011.196 PMC3193543

[30] Bikle, D. D. , Y. Oda , C. L. Tu and Y. Jiang (2015) Novel mechanisms for the vitamin D receptor (VDR) in the skin and in skin cancer. J. Steroid Biochem. 148, 47–51. 10.1016/j.jsbmb.2014.10.017 PMC4361259 25445917

[31] Bikle, D. D. , Y. Jiang , T. Nguyen , Y. Oda and C. L. Tu (2016) Disruption of vitamin D and calcium signaling in keratinocytes predisposes to skin cancer. Front. Physiol. 7, 296. 27462278 10.3389/fphys.2016.00296 PMC4940389

[32] Nemeth, E. F. , E. G. Delmar , W. L. Heaton , M. A. Miller , L. D. Lambert , R. L. Conklin , M. Gowen , J. G. Gleason , P. K. Bhatnagar and J. Fox (2001) Calcilytic compounds: potent and selective Ca^2+^ receptor antagonists that stimulate secretion of parathyroid hormone. J. Pharmacol. Exp. Ther. 299, 323–331. 11561095

[33] McLeod, S. D. , C. Smith and R. S. Mason (1995) Stimulation of tyrosinase in human melanocytes by pro‐opiomelanocortin‐derived peptides. J. Endocrinol. 146, 439–447. 7595139 10.1677/joe.0.1460439

[34] Rybchyn, M. S. , W. G. M. De Silva , V. B. Sequeira , B. Y. McCarthy , A. V. Dilley , K. M. Dixon , G. M. Halliday and R. S. Mason (2018) Enhanced repair of UV‐induced DNA damage by 1,25‐dihydroxyvitamin D‐3 in skin is linked to pathways that control cellular energy. J. Investigat. Dermatol. 138, 1146–1156. 10.1016/j.jid.2017.11.037 29258892

[35] Sequeira, V. B. , M. S. Rybchyn , C. Gordon‐Thomson , W. Tongkao‐on , M. T. Mizwicki , A. W. Norman , V. E. Reeve , G. M. Halliday and R. S. Mason (2013) Opening of chloride channels by 1alpha,25‐dihydroxyvitamin D3 contributes to photoprotection against UVR‐induced thymine dimers in keratinocytes. J. Invest. Dermatol. 133, 776–782. 23014341 10.1038/jid.2012.343

[36] Reeve, V. E. , D. Domanski and M. Slater (2006) Radiation sources providing increased UVA/UVB ratios induce photoprotection dependent on the UVA dose in hairless mice. Photochem. Photobiol. 82, 406–411. 16613492 10.1562/2005-09-29-RA-703

[37] Rybchyn, M. S. , M. Slater , A. D. Conigrave and R. S. Mason (2011) An Akt‐dependent increase in canonical Wnt signaling and a decrease in sclerostin protein levels are involved in strontium ranelate‐induced osteogenic effects in human osteoblasts. J. Biol. Chem. 286, 23771–23779. 21566129 10.1074/jbc.M111.251116 PMC3129158

[38] Honigsmann, H. , W. Brenner , A. Tanew and B. Ortel (1987) UV‐induced unscheduled DNA synthesis in human skin: Dose response, correlation with erythema, time course and split dose exposure in vivo. J. Photochem. Photobiol. B 1, 33–43. 3149981 10.1016/1011-1344(87)80004-0

[39] Rasmussen, R. E. and R. B. Painter (1964) Evidence for repair of ultra‐violet damaged deoxyribonucleic acid in cultured mammalian cells. Nature 203, 1360–1362. 14207310 10.1038/2031360a0

[40] Demetriou, S. K. , K. Ona‐Vu , A. E. Teichert , J. E. Cleaver , D. D. Bikle and D. H. Oh (2012) Vitamin D receptor mediates DNA repair and is UV inducible in intact epidermis but not in cultured keratinocytes. J. Invest. Dermatol. 132, 2097–2100. 22495177 10.1038/jid.2012.107 PMC3396713

[41] Bikle, D. D. , Z. Xie and C. L. Tu (2012) Calcium regulation of keratinocyte differentiation. Expert Rev. Endocrinol. Metabol. 7, 461–472. 10.1586/eem.12.34 PMC3491811 23144648

[42] Courdavault, S. , C. Baudouin , M. Charveron , B. Canguilhem , A. Favier , J. Cadet and T. Douki (2005) Repair of the three main types of bipyrimidine DNA photoproducts in human keratinocytes exposed to UVB and UVA radiations. DNA Repair 4, 836–844. 15950551 10.1016/j.dnarep.2005.05.001

[43] Birch‐Machin, M. A. , E. V. Russell and J. A. Latimer (2013) Mitochondrial DNA damage as a biomarker for ultraviolet radiation exposure and oxidative stress. Br. J. Dermatol. 169(Suppl 2), 9–14. 23786615 10.1111/bjd.12207

[44] Lamola, A. A. (1971) Production of pyrimidine dimers in DNA in the dark. Biochem. Biophys. Res. Comm. 43, 893–898. 4935292 10.1016/0006-291x(71)90701-7

[45] Mouret, S. , C. Baudouin , M. Charveron , A. Favier , J. Cadet and T. Douki (2006) Cyclobutane pyrimidine dimers are predominant DNA lesions in whole human skin exposed to UVA radiation. Proc. Natl Acad. Sci. 103, 13765–13770. 16954188 10.1073/pnas.0604213103 PMC1564232

[46] Tu, C. L. , A. Celli , T. Mauro and W. Chang (2019) Calcium‐sensing receptor regulates epidermal intracellular Ca(2+) signaling and re‐epithelialization after wounding. J. Invest. Dermatol. 139, 919–929. 30404020 10.1016/j.jid.2018.09.033 PMC6431556

[47] Dhanalakshmi, S. , G. U. Mallikarjuna , R. P. Singh and R. Agarwal (2004) Silibinin prevents ultraviolet radiation‐caused skin damages in SKH‐1 hairless mice via a decrease in thymine dimer positive cells and an up‐regulation of p53–p21/Cip1 in epidermis. Carcinogenesis 25 (8):1459–1465. 15033902 10.1093/carcin/bgh152

[48] Aggarwal, A. , M. Prinz‐Wohlgenannt , S. Tennakoon , J. Hobaus , C. Boudot , R. Mentaverri , E. M. Brown , S. Baumgartner‐Parzer and E. Kallay (2015) The calcium‐sensing receptor: A promising target for prevention of colorectal cancer. Biochim. Biophys. Acta 1853 (9):2158–2167. 25701758 10.1016/j.bbamcr.2015.02.011 PMC4549785

[49] Anderson, A. , A. Bowman , S. J. Boulton , P. Manning and M. A. Birch‐Machin (2014) A role for human mitochondrial complex II in the production of reactive oxygen species in human skin. Redox Biol. 2, 1016–1022. 25460738 10.1016/j.redox.2014.08.005 PMC4215388

[50] Javeri, A. , X. X. Huang , F. Bernerd , R. S. Mason and G. M. Halliday (2008) Human 8‐oxoguanine‐DNA glycosylase 1 protein and gene are expressed more abundantly in the superficial than basal layer of human epidermis. DNA Repair 7, 1542–1550. 18585103 10.1016/j.dnarep.2008.05.011

[51] Rinnerthaler, M. , J. Bischof , M. K. Streubel , A. Trost and K. Richter (2015) Oxidative stress in aging human skin. Biomolecules 5, 545–589. 25906193 10.3390/biom5020545 PMC4496685

[52] Wong, C. T. and D. H. Oh (2021) Vitamin D receptor promotes global nucleotide excision repair by facilitating XPC dissociation from damaged DNA. J. Invest. Dermatol. 141, 1656–1663. 33524369 10.1016/j.jid.2020.11.033

[53] Yiu, A. J. , C. L. Ibeh , S. K. Roy and B. C. Bandyopadhyay (2017) Melamine induces Ca2+‐sensing receptor activation and elicits apoptosis in proximal tubular cells. Am. J. Physiol‐cell Ph. 313, C27–C41. 10.1152/ajpcell.00225.2016 PMC5538798 28381520

[54] Gutierrez‐Lopez, T. Y. , L. B. Orduna‐Castillo , M. N. Hernandez‐Vasquez , J. Vazquez‐Prado and G. Reyes‐Cruz (2018) Calcium sensing receptor activates the NLRP3 inflammasome via a chaperone‐assisted degradative pathway involving Hsp70 and LC3‐II. Biochem. Biophys. Res. Comm. 505, 1121–1127. 30316511 10.1016/j.bbrc.2018.10.028

[55] Lee, G.‐S. , N. Subramanian , A. I. Kim , I. Aksentijevich , R. Goldbach‐Mansky , D. B. Sacks , R. N. Germain , D. L. Kastner and J. J. Chae (2012) The calcium‐sensing receptor regulates the NLRP3 inflammasome through Ca^2+^ and cAMP. Nature 492, 123‐+. 23143333 10.1038/nature11588 PMC4175565

[56] Rossol, M. , M. Pierer , N. Raulien , D. Quandt , U. Meusch , K. Rothe , K. Schubert , T. Schoneberg , M. Schaefer , U. Krugel , S. Smajilovic , H. Brauner‐Osborne , C. Baerwald and U. Wagner (2012) Extracellular Ca2+ is a danger signal activating the NLRP3 inflammasome through G protein‐coupled calcium sensing receptors. Nat. Commun. 3. 10.1038/ncomms2339 PMC3535422 23271661

[57] Zhai, T. Y. , B. H. Cui , L. Zou , J. Y. Zeng , S. Gao , Q. Zhao , Y. Wang , W. L. Xie and Y. H. Sun (2017) Expression and role of the calcium‐sensing receptor in rat peripheral blood polymorphonuclear neutrophils. Oxid. Med Cell Longev. 2017, 1–10. 10.1155/2017/3869561 PMC5610836 29081886

[58] Li, T. T. , M. R. Sun , X. Yin , C. L. Wu , Q. Y. Wu , S. L. Feng , H. Li , Y. Luan , J. Wen , L. X. Yan , B. H. Zhao , C. Q. Xu and Y. H. Sun (2013) Expression of the calcium sensing receptor in human peripheral blood T lymphocyte and its contribution to cytokine secretion through MAPKs or NF‐kappa B pathways (vol 53, pg 414, 2013). Mol. Immunol. 55, 429. 10.1016/j.molimm.2012.09.010 23103379

[59] Wu, C.‐L. , Q.‐Y. Wu , J.‐J. Du , J.‐Y. Zeng , T.‐T. Li , C.‐Q. Xu and Y.‐H. Sun (2015) Calcium‐sensing receptor in the T lymphocyte enhanced the apoptosis and cytokine secretion in sepsis. Mol. Immunol. 63, 337–342. 25256599 10.1016/j.molimm.2014.08.007

[60] Hu, B. , F. Tong , L. Xu , Z. Shen , L. Yan , G. Xu and R. Shen (2018) Role of calcium sensing receptor in streptozotocin‐induced diabetic rats exposed to renal ischemia reperfusion injury. Kidney Blood Press Res. 43, 276–286. 29490306 10.1159/000487685

[61] Chen, Y. , X. Li , X. Gan , J. Qi , B. Che , M. Tai , S. Gao , W. Zhao , N. Xu and Z. Hu (2019) Fucoidan from *Undaria pinnatifida* ameliorates epidermal barrier disruption via keratinocyte differentiation and CaSR level regulation. Mar Drugs 17. 10.3390/md17120660 PMC6950751 31771286

[62] Garmyn, M. , A. R. Young and S. A. Miller (2018) Mechanisms of and variables affecting UVR photoadaptation in human skin. Photochem. Photobiol. Sci. 17, 1932–1940. 29926025 10.1039/c7pp00430c

[63] Breitwieser, G. E. (2013) The calcium sensing receptor life cycle: trafficking, cell surface expression, and degradation. Best Pract. Res. Clin. Endocrinol. Metab. 27, 303–313. 23856261 10.1016/j.beem.2013.03.003

[64] Conigrave, A. D. and D. T. Ward (2013) Calcium‐sensing receptor (CaSR): pharmacological properties and signaling pathways. Best Pract. Res. Clin. Endocrinol. Metab. 27, 315–331. 23856262 10.1016/j.beem.2013.05.010

[65] Broadhead, G. K. , H. C. Mun , V. A. Avlani , O. Jourdon , W. B. Church , A. Christopoulos , L. Delbridge and A. D. Conigrave (2011) Allosteric modulation of the calcium‐sensing receptor by gamma‐glutamyl peptides: inhibition of PTH secretion, suppression of intracellular cAMP levels, and a common mechanism of action with L‐amino acids. J. Biol. Chem. 286, 8786–8797. 21187282 10.1074/jbc.M110.149724 PMC3059007

[66] Centeno, P. P. , A. Herberger , H. C. Mun , C. Tu , E. F. Nemeth , W. Chang , A. D. Conigrave and D. T. Ward (2019) Phosphate acts directly on the calcium‐sensing receptor to stimulate parathyroid hormone secretion. Nat. Commun. 10, 4693. 31619668 10.1038/s41467-019-12399-9 PMC6795806

[67] Riccardi, D. , M. Traebert , D. T. Ward , B. Kaissling , J. Biber , S. C. Hebert and H. Murer (2000) Dietary phosphate and parathyroid hormone alter the expression of the calcium‐sensing receptor (CaR) and the Na+‐dependent Pi transporter (NaPi‐2) in the rat proximal tubule. Pflugers Arch. 441, 379–387. 11211126 10.1007/s004240000436

[68] Chattopadhyay, N. , G. Legradi , M. Bai , O. Kifor , C. Ye , P. M. Vassilev , E. M. Brown and R. M. Lechan (1997) Calcium‐sensing receptor in the rat hippocampus: A developmental study. Brain Res. Dev. Brain Res. 100, 13–21. 9174241 10.1016/s0165-3806(97)00009-6

[69] Riccardi, D. , A. E. Hall , N. Chattopadhyay , J. Z. Xu , E. M. Brown and S. C. Hebert (1998) Localization of the extracellular Ca^2+^/polyvalent cation‐sensing protein in rat kidney. Am. J. Physiol. 274, F611–622. 9530279 10.1152/ajprenal.1998.274.3.F611

[70] Bruce, J. I. , X. Yang , C. J. Ferguson , A. C. Elliott , M. C. Steward , R. M. Case and D. Riccardi (1999) Molecular and functional identification of a Ca^2+^ (polyvalent cation)‐sensing receptor in rat pancreas. J. Biol. Chem. 274, 20561–20568. 10400686 10.1074/jbc.274.29.20561

[71] Leach, K. , K. J. Gregory , I. Kufareva , E. Khajehali , A. E. Cook , R. Abagyan , A. D. Conigrave , P. M. Sexton and A. Christopoulos (2016) Towards a structural understanding of allosteric drugs at the human calcium‐sensing receptor. Cell Res. 26, 574–592. 27002221 10.1038/cr.2016.36 PMC4856764

[72] Miedlich, S. U. , L. Gama , K. Seuwen , R. M. Wolf and G. E. Breitwieser (2004) Homology modeling of the transmembrane domain of the human calcium sensing receptor and localization of an allosteric binding site. J. Biol. Chem. 279, 7254–7263. 14660633 10.1074/jbc.M307191200

