# Supplementary material for: UV‐induced DNA Damage in Skin is Reduced by CaSR Inhibition
Source: Photochem Photobiol. 2022 Mar 29;98(5):1157–66. doi: 10.1111/php.13615 (PMC9540002; doi:10.1111/php.13615)
Supplement: Supplementary file 1 — Figure S1. NPS‐2143 promoted repair. Figure S2. NPS‐2143 and 1,25(OH)2D3 enhanced cell survival after UV. Figure S3. Tunicamycin reduced expression of CaSR at the plasma membrane. Figure S4. Tunicamycin pre‐treatment had no effect on NPS‐2143 suppression of UV‐induced 8‐OHdG. Table S1. siRNA duplex sequences obtained from Santa Cruz Biotechnology. [file PHP-98-1157-s001.docx]

# SUPPORTING INFORMATION

**UV-induced DNA Damage in Skin is Reduced by CaSR Inhibition**

Chen Yang^1^, Mark Stephen Rybchyn^1, 2^, Warusavithana Gunawardena Manori De Silva^1^, Jim Matthews^3^, Andrew J.A. Holland^4^, Arthur David Conigrave^5^, Rebecca Sara Mason^1, 5^

^1^ Department of Physiology and Bosch Institute, School of Medical Sciences, University of Sydney, New South Wales 2006, Australia_;_ ^2^ School of Chemical Engineering, University of New South Wales, Sydney, NSW 2033, Australia_;_  ^3^ Sydney Informatics Hub, University of Sydney, New South Wales, Australia_;_  ^4^ Douglas Cohen Department of Paediatric Surgery, The University of Sydney School of Medicine, The Children’s Hospital at Westmead Clinical School, Faculty of Medicine and Health, Sydney, New South Wales, Australia_;_  ^5^School of Life and Environmental Sciences, Charles Perkins Centre (D17), University of Sydney, New South Wales 2006, Australia

*Corresponding author email: Rebecca Sara Mason, University of Sydney, NSW 2006, Australia. E-mail: rebecca.mason@sydney.edu.au

#

# Section S1. Materials and Methods

## Cell Titer-Blue® Cell Viability Assay

Cell viability was determined using the Cell Titer-Blue® Cell Viability Assay (Promega) according to the manufacturer’s instructions. The fluorescence signal (Excitation/Emission=560nm/590nm) was read by CLARIOstar® Multimode Microplate Reader (BMG Labtech).

## Tunicamycin pretreatment

Tunicamycin (Sigma-Aldrich), an N-linked glycosylation inhibitor^73, 74^ , was added into the medium at 1 (μg/ml) and incubated with cells under normal growth condition for 48hrs.

## CaSR immuno-fluorescence on non-permeabilized cells

Cells were plated on glass coverslips (13mm) in 24 well plates. Tunicamycin-treated or DMSO-treated cells were fixed with 4% paraformaldehyde in PBS for 10 minutes without any subsequent cell permeabilization, based on previously published methods ^75, 76^. At room temperature, the cells were blocked with 5% horse serum in PBS for 1h, followed by incubation with primary anti-CaSR mouse antibody (HL1499) at 1 μg/ml in 0.1% Tween Tris-Buffered Saline for 1h in a 37°C humidified incubator. The HL1499 antibody used in the current study recognizes residues 15-29 of the N-terminal extracellular domain, which does not contain any glycosylation residues, and detects both glycosylated and un-glycosylated isoforms ^77^. Isotype control used mouse lgG instead of primary antibody. After two 15-minute washes with PBS, the cells were incubated with FITC-conjugated sheep anti-mouse IgG (Ex 493nm/Em528nm, Silenus Laboratories, 1/500 in PBS) for 1 hour at room temperature, followed by four rounds of 15-minute washes. The coverslip was mounted with UltraCruz™ Mounting Medium (Santa Cruz) containing 4',6-diamidino-2-phenylindole (DAPI) to visualize the nuclei and images were then acquired with a Zeiss LSM800 confocal microscope (Zeiss).

**
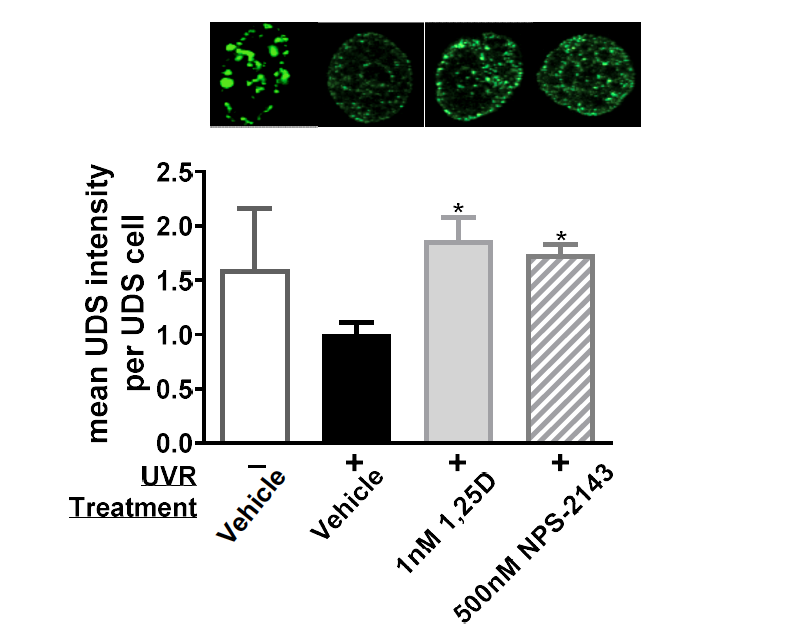
**

Figure S1. NPS-2143 promoted repair.

Incorporation of thymidine analog EdU after 90mins with or without treatments in the nucleus of keratinocytes (DAPI counterstained) was imaged under confocal microscope. EdU positive cells were shown with green punctate staining. Average intensity of EdU incorporation per UDS positive cell were measured as Means + SEM (10 nuclei measured per coverslip) with example micrograph of single Edu positive cells, scale bar = 2 µm. Results were from two experiments, each performed in triplicate (n=6). *p<0.05, when compared with UV+Vehicle.


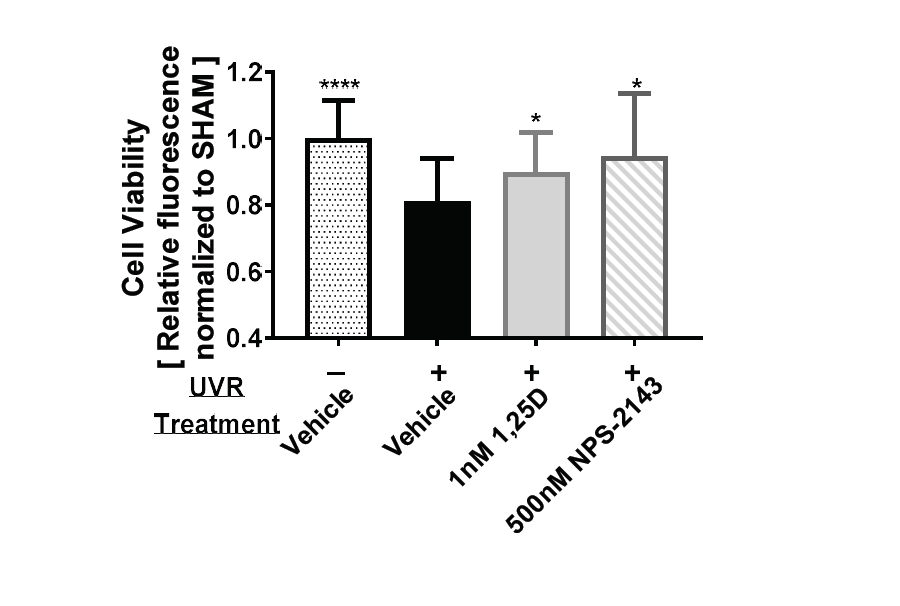


Figure S2. NPS-2143 and 1,25(OH)_2_D_3_ enhanced cell survival after UV.

Keratinocytes plated in 96 well plates were irradiated with 400 mJ/cm2 UVB then treated with vehicle, 1 nM 1,25(OH)2D3 or NPS-2143 in the presence of 1mM CaCl_2_ for 3hrs. The fluorescence values from Titer-Blue® Cell Viability Assay were all normalized to SHAM. Cell viability was shown as Mean +SD. Results are pooled from 5 experiments each with triplicates. ***p<0.001, * p<0.5 compared with UV+vehicle.


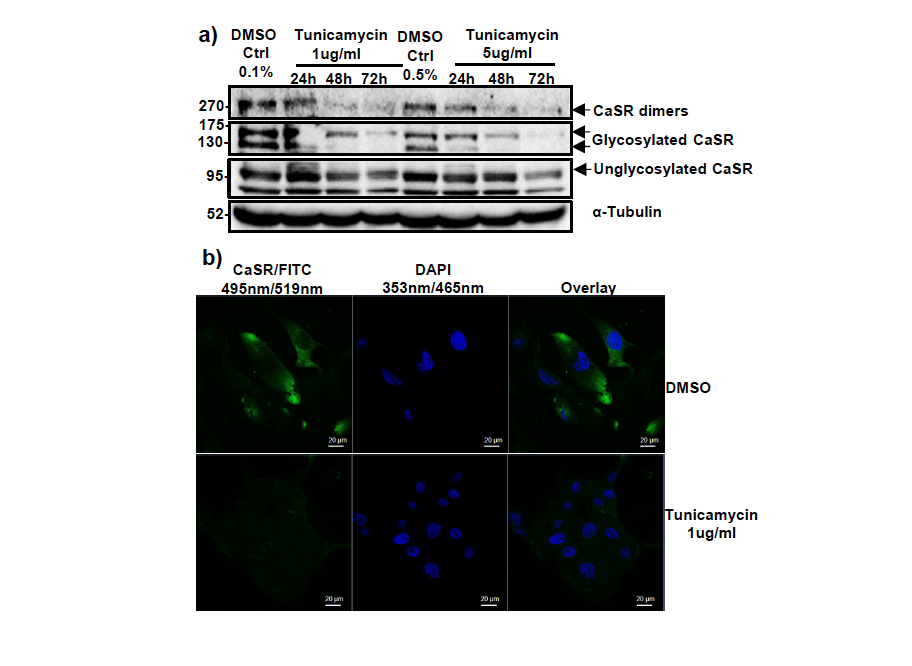


Figure S3. Tunicamycin reduced expression of CaSR at the plasma membrane.

**a)** **CaSR expression by Western blot.** Individual sections of this western blot membrane (separated by solid lines) were exposed for different periods (224.5 s /46.3 s /22.1 s) shown in separate panels from top) to visualize CaSR isoforms at different sizes that were present in the cell (n=3). **b)** **Confocal microscopy images of CaSR** (Green) after tunicamycin (1 µg/ml) treatment for 48 h in keratinocytes, counterstained by DAPI (Blue) (n=2), scale bar = 20 µm


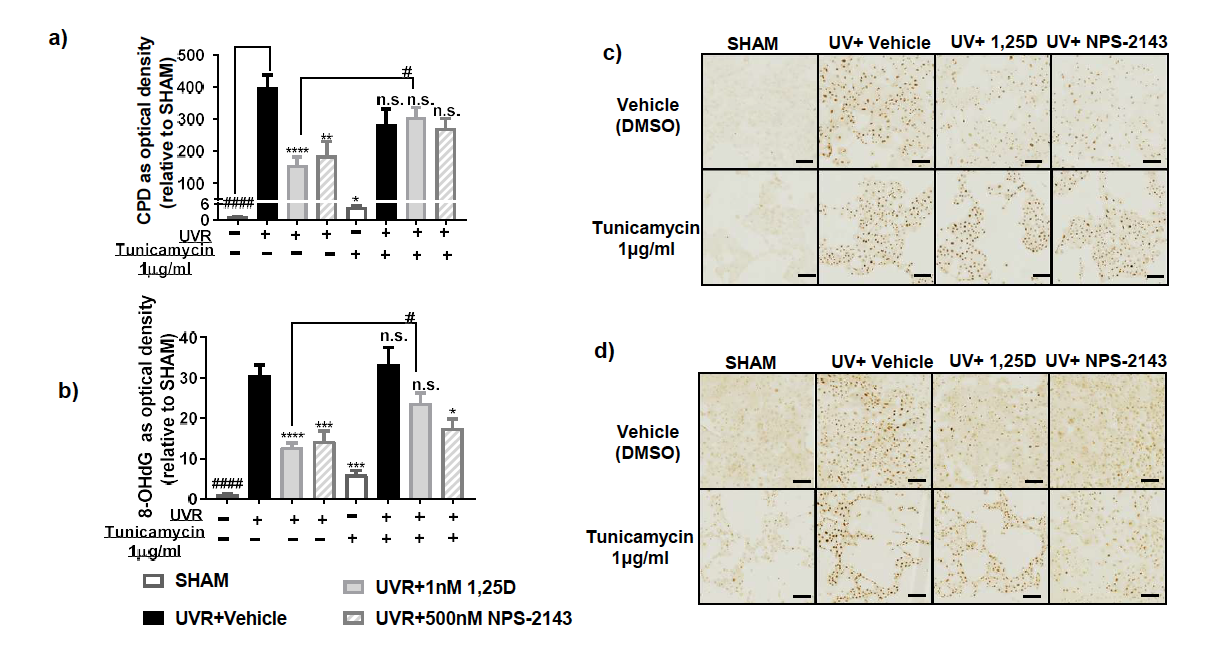


Figure S4. Tunicamycin pre-treatment had no effect on NPS-2143 suppression of UV-induced 8-OHdG.

a) CPD or b) 8-OHdG (y-axis) by immunohistochemistry and image analysis, shown as Means +SEM (n=9). Keratinocytes were treated with 1 ug/ml tunicamycin or vehicle (DMSO) for 48 h, followed by UV exposure and then addition of ethanol control or 500 nM NPS-2143 or 1 nM 1,25(OH)_2_D_3_ for 3h in the presence of 1mM Ca^2+^. *****p<*0.0001, ***p*<0.01, **p*<0.05, and n.s. not significant compared with UV+Vehicle (non tunicamycin treated); ####, p<0.001 significantly different from UV+Vehicle (non-tunicamycin treated). Photomicrographs of c) CPD and d) 8-OHdG in human primary keratinocytes with or without treatment with tunicamycin. Scale bar = 100μm

**Table S1. siRNA duplex sequences obtained from Santa Cruz Biotechnology.**

| **Gene** | **manufacturer code** | **siRNA duplex sequence 5’to 3’** |
| --- | --- | --- |
| CaSR (Human) | A | Sense: CUGGUUACAGGCUAUGAUAtt  Antisense: UAGUACUGCAUUUGCUUGGtt |
|  | B | Sense: CUUGCAACACCGUUUCUAAtt  Antisense:UUAGAAACGGUGUUGCAAGtt |
|  | C | Sense: GAAUGUAUCUCCUCCUAUUtt  Antisense: AAUAGGAGGAGAUACAUUCtt |

References:

73 Fan, G., P. K. Goldsmith, R. Collins, C. K. Dunn, K. J. Krapcho, K. V. Rogers and A. M. Spiegel (1997) N‐linked glycosylation of the human Ca^2+^ receptor is essential for its expression at the cell surface. Endocrinology 138, 1916–1922.

74 Huang, Y., J. Niwa, G. Sobue and G. E. Breitwieser (2006) Calcium‐sensing receptor ubiquitination and degradation mediated by the E3 ubiquitin ligase dorfin. J. Biol. Chem. 281, 11610–11617.

75 Tharmalingam, S., A. M. Daulat, J. E. Antflick, S. M. Ahmed, E. F. Nemeth, S. Angers, A. D. Conigrave and D. R. Hampson (2011) Calcium‐sensing receptor modulates cell adhesion and migration via integrins. J. Biol. Chem. 286, 40922–40933.

76 Bouschet, T., S. Martin and J. M. Henley (2005) Receptor‐activity‐modifying proteins are required for forward trafficking of the calcium‐sensing receptor to the plasma membrane. J. Cell Sci. 118, 4709–4720.

77 Handlogten, M. E., C. Huang, N. Shiraishi, H. Awata and R. T. Miller (2001) The Ca2+‐sensing receptor activates cytosolic phospholipase A2 via a Gqalpha ‐dependent ERK‐independent pathway. J. Biol. Chem. 276, 13941–13948.
